# Microwave Optimized Synthesis of *N*-(adamantan-1-yl)-4-[(adamantan-1-yl)-sulfamoyl]benzamide and Its Derivatives for Anti-Dengue Virus Activity

**DOI:** 10.3390/molecules23071678

**Published:** 2018-07-10

**Authors:** Jacques Joubert, Eugene B. Foxen, Sarel F. Malan

**Affiliations:** Pharmaceutical Chemistry, School of Pharmacy, University of the Western Cape, Bellville 7530, South Africa; efoxen@gmail.com (E.B.F.); sfmalan@uwc.ac.za (S.F.M.)

**Keywords:** amantadine, sulfonamide, microwave irradiation, anti-dengue virus serotype 2 activity, cytotoxicity, docking

## Abstract

Dengue fever is a major public health concern in many tropical and sub-tropical regions. The development of agents that are able to inhibit the dengue virus (DENV) is therefore of utmost importance. This study focused on the synthesis of dual acting hybrids comprising structural features of known DENV inhibitors, amantadine (**1**) and benzsulfonamide derivatives. Hybrid compound **3**, *N*-(adamantan-1-yl)-4-[(adamantan-1-yl)sulfamoyl]benzamide, was synthesized by reacting amantadine (**1**) with 4-(chlorosulfonyl)benzoic acid (**2**), after optimization, in a 2:1 ratio under microwave irradiation conditions in a one-pot reaction. Mono-adamantane derivatives **6** and **7** were synthesised via acyl halide formation of benzoic acid (**4**) and 4-sulfamoyl benzoic acid (**5**), respectively, followed by conjugation with amantadine (**1**) through a conventional or microwave irradiation assisted nucleophilic addition/substitution reaction. The use of microwave irradiation lead to significant increases in yields and a reduction in reaction times. Nuclear magnetic resonance, infra-red and mass spectral data confirmed the structures. Compound **3** and **7** showed significant anti-DENV serotype 2 activity (IC_50_ = 22.2 µM and 42.8 µM) and low cytotoxicity (CC_50_ < 100 µM). Possible mechanisms of action are also proposed, which are based on the biological results and molecular docking studies.

## 1. Introduction

Dengue fever is a rapidly spreading mosquito-borne viral illness affecting human populations in tropical and subtropical regions. In recent years, there has been an increase in the incidence of the disease, from nine dengue-reporting countries in the 1950’s to a current public health concern in over 100 countries [1]. The dengue virus (DENV) exists as four closely related serotypes, DENV1, DENV2, DENV3, and DENV4. Infection with one serotype leads to the self-limiting less severe dengue fever, whereas infection with multiple serotypes may precipitate the more serious life-threatening dengue hemorrhagic fever and dengue shock syndrome [2]. Worldwide more than 390 million people become infected with the DENV each year, with approximately 20,000 deaths [1]. The lack of an effective vaccine or approved chemotherapeutic agent for the dengue disease has prompted urgent investigations into these areas to enable adequate prevention and treatment. Given the growing global burden that is imposed by the DENV, intensified efforts to investigate new effective scaffolds as potential inhibitors of the DENV are warranted.

**Figure 1:**
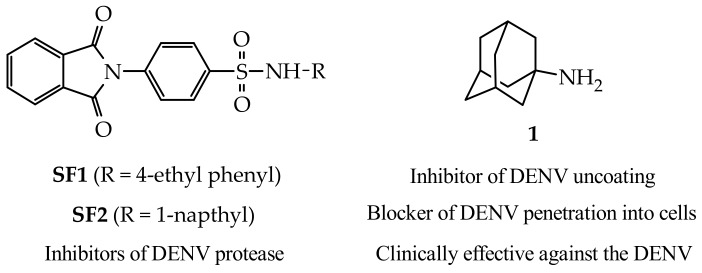
Benzsulfonamide derivatives (**SF1** and **SF2**) as DENV2 NS2B/NS3 protease inhibitors and the structure of the anti-DENV agent, amantadine (**1**).

The various stages in the DENV replication cycle are all potential targets for the inhibition of the virus [3]. One promising target to prevent viral replication is to inhibit the non-structural protein DENV NS2B/NS3 protease. The NS2B/NS3 pro-complex helps to cleave viral proteins to perform their functions and any disturbance in functional behavior of this region is reported to inhibit viral replication [2,4,5,6,7]. A study done by Timiri et al., (2015) [6], demonstrated in vitro, the ability of benzsulfonamide derivatives, as represented by **SF1** and **SF2** in Figure 1, to inhibit the dengue virus serotype 2 (DENV2) NS2B/NS3 protease (IC_50_ = 48.2 µM and 121.9 µM, respectively). Amantadine (**1**, Figure 1), a polycyclic cage compound, has shown anti-DENV activity on all four serotypes in vitro via a mechanism that is speculated to involve inhibition of the uncoating step in the virus life cycle and/or blocking virus penetration into the host cell (Koff et al., 1980) [8]. A more recent small scale clinical study by Lin and Chen, (2016) [9] indicated that amantadine is an effective treatment option in vivo against DENV infected patients. In addition, amantadine has also shown the ability to improve the activity and distribution profiles of compounds it is conjugated to, making it an excellent scaffold for use in drug design [10].

**Figure 2:**
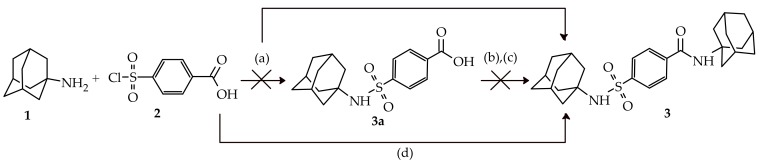
Initial proposed synthesis route for **3** via intermediate **3a** (steps a–c). Actual one step synthesis of compound **3** either through step (a) or optimized step (d). Reagents and conditions: (a) 1:1 ratio of **1** and 2, 25 °C, CHCl_3_, triethylamine, 48 h; (b) reflux, DMF, CH_2_Cl_2_, thionyl chloride, 2 h; (c) 25 °C, 1 eq amantadine (**1**), CHCl_3_, triethylamine, until completion; and, (d) 2:1 ratio of **1** and **2**, MW, 100 °C, 150 W, 250 psi, CHCl_3_, triethylamine, 30 min.

**Figure 3:**
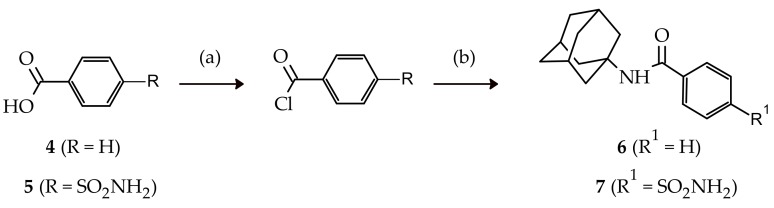
Synthesis of compound **6** and **7**. Reagents and conditions: (a) reflux, DMF, CH_2_Cl_2_, thionyl chloride, 2 h; (b) 25 °C, 1 eq amantadine (**1**), CHCl_3_, trietylamine, 2 h or MW, 100 °C, 150 W, 250 psi, 30 min.

The rationale behind this current study was to synthesise dual acting hybrid molecules, with each portion of the hybrid possessing intrinsic anti-DENV activity. The intention was thus to develop adamantane-benzsulfonamide hybrids with greater antiviral activity than their individual parent compounds, i.e., amantadine (**1**) and benzsulfonamide derivatives (**SF1** and **SF2**). Here, we describe the optimized synthesis, preliminary in vitro screening, and molecular modelling studies of three hybrid molecules (compounds **3**, **6**, and **7**, Figure 2 and Figure 3) against the DENV2.

## 2. Results and Discussion

### 2.1. Chemistry

The initial synthesis route of novel compound **3** (Figure 2, steps a–c) was developed to first produce the mono-substituted adamantane derivative 4-[(adamantan-1-yl)sulfamoyl]benzoic acid (**3a**) via sulfonamide formation between **1** and **2**, followed by acyl halide formation of **3a** and subsequent conjugation with amantadine (**1**). In the initial reaction, a 1:1 ratio of starting materials **1** and **2** dissolved in CHCl_3_ were reacted at room tempreture for 48 h. According to thin-layer chromatography analysis, the majority of the newly formed compound was located at a retardation factor (Rf) value (Rf = 0.8, hexane:ethyl acetate, 70:30) that was not expected for the desired compound (**3a**, expected Rf between 0.1–0.3). Isolation via column chromatography of the newly formed compound followed by NMR and MS analysis, as discussed below, indicated that the desired dimeric adamantane compound *N*-(adamantan-1-yl)-4-[(adamantan-1-yl)sulfamoyl]benzamide (**3**) was obtained in 25% yield.

In an attempt to optimize the reaction of **3**, a two-fold excess of **1** was used and reacted under microwave irradiation conditions (100 °C, 150 W, 250 psi) for 30 minutes to produce **3** in a good yield of 78% (Figure 2). The microwave irradiation reaction lead to a marked decrease in reaction time and improved the yield when compared to the conventional method (Table 1). The ability to synthesise compound **3** in a one-pot reaction using both the conventional and microwave assisted methods may be explained by the in-situ formation of the 4-(chlorosulfonyl)benzoic anhydride intermediate and subsequent nucleophilic substitution with **1** to give the amide. This is similar to a mechanism described previously [11]. The chlorosulfonyl function will then be free to undergo sulfonamide conjugation with **1** to give compound **3**. However, further studies are necessary to closely monitor this reaction utilising NMR methods in order to propose an accurate mechanism of action.

In order to develop preliminary structure activity relationships of **3**, mono-adamantane substituted *N*-(adamantan-1-yl)benzamide (**6**), and N-(adamantan-1-yl)-4-sulfamoylbenzamide (**7**) were synthesised (Figure 3). These compounds were synthesised via acyl halide formation of starting materials benzoic acid (**4**) and 4-sulfamoyl benzoic acid (**5**), respectively, followed by conjugation with amantadine (**1**) through a conventional nucleophilic addition/substitution reaction in moderate yields (25% and 40%, respectively). The nucleophilic addition/substitution reaction step was also conducted under microwave irradiation, leading to improved yields (68% and 82%, respectively) and significantly reduced reaction times (Table 1). Compound **6** is commercially available from numerous chemical vendors, however the microwave synthesis method reported here is novel.

All of the compounds were characterized by ^1^H-, ^13^C-nuclear magnetic resonance (NMR), infra-red (IR), and mass spectra. The structure of the adamantane moiety was one common feature in all of the synthesised compounds. This structural component had characteristic ^1^H-NMR and ^13^C-NMR signal patterns, which were used unequivocally to establish the presence of the adamantane component in the final compounds.

In the ^1^H-NMR of compound **3** the thirty protons of the two adamantane moieties were observed between 2.13 and 1.53 as a series of singlets, doublets, and multiplets. For both compounds **6** and **7**, the protons of the adamantane moiety was observed between 2.1–1.6 ppm integrating for the required fifteen hydrogens with a similar splitting pattern, as previously described for related compounds [12]. The *para* substituted aromatic protons of compound **3** showed two doublets at δ 7.93 and 7.82 ppm with *J* = 8.4 Hz, indicating a strong coupling from their respective *ortho* protons. A similar strong coupling was observed for compound **7** for the two doublets observed at 7.85 and 7.77 ppm with *J* = 8.7 Hz. Upon closer inspection the two doublets of **3** and **7**, respectively, appear as apparent doublet of triplets with *J* = 2 Hz of each triplet. This substantiates the long-range coupling of each of the aromatic protons by their respective meta and para protons apart from strong coupling from their ortho protons. The aromatic protons of compound **6** was observed as two multiplets between 7.72–7.38 ppm integrating for the required five hydrogens.

In the ^13^C-NMR, the aliphatic carbon signals of the adamantane compound had four distinct shifts. For instance, the chemical shift of the quaternary adamantane carbon ranged from δ 55 to δ 52 ppm and the three symmetrical CH adamantane groups showed a distinct signal in the proximity of δ 41 ppm. The two separate pairs of three symmetrical CH_2_ adamantane groups were observed in the area of δ 36 and δ 29 ppm, respectively. For both compounds **6** and **7**, the four distinct adamantane carbon signals were observed. However, for compound **3**, eight carbon signals in close proximity to each other were observed because of the two differently conjugated adamantane structures. This observation further confirmed the dimeric adamantane nature of compound **3**. The aromatic carbon signals of the synthesised compounds were present between δ 165 and δ 125 ppm. The amide carbonyl signal for all three compounds were observed between δ 167 to δ 165 ppm.

In addition to the above mentioned distinctive NMR signals, the IR spectra of these adamantane containing compounds had a typical IR absorption for sp^3^ hybridised C-H. The absorption for C-H stretching vibration showed two strong characteristic peaks at ≈2910 cm^−1^ and ≈2850 cm^−1^. The IR spectra also showed an intense absorption peak in the region of 1720–1630 cm^−1^ because of the amide C=O present in compounds **3**, **6** and **7**. A sharp aromatic sulphonamide peak was present for compounds **3** and **7** in the region of 3345 cm^−1^. The mass spectral data confirmed the molecular weights of the final compounds. In addition, the high resolution mass spectra of compounds **3**, **6**, and **7** showed an error of less than 0.004 *m*/*z* units from the calculated mass, thus confirming their high purity.

### 2.2. In-Vitro Anti-DENV2 Activity and Cytotoxicity Evaluations

The antiviral activity of the test compounds (**1**, **3**, **6**, and **7**) was examined in the Cell-based Flavivirus Immuno-assay (CFI assay). The CFI assay is based on a quantitative immunodetection of the DENV envelope protein production in A549 cells [13]. Infection of the A549 cells by the DENV2 was allowed to occur in the presence of each test compound, after which virus load was measured by quantifying the amount of envelope protein that is produced using enzyme linked immunosorbent assay (ELISA). To ensure that the inhibition of the envelope production was not due to cell death, the cytotoxicity of each test compound was assessed via the cell viability assay [13]. Results from these assays are presented in Table 2.

Compound **3** exhibited the best anti-DENV2 activity in this assay (IC_50_ = 22.4 ± 7.7 µM), which was approximately two-fold that of the structurally related compound **7** (IC_50_ = 42.8 ± 8.6 µM). This indicates that the di-adamantane substitution is favoured for DENV2 inhibition, and may suggest a synergistic effect of the two adamantane moieties. Compound **6**, which is devoid of the sulfonamide function of compounds **3** and **7**, did not show any significant activity (IC_50_ > 100 µM) substantiating the importance of the sulfonamide moiety in these structures to possess anti-DENV activity in this assay. Amantadine (**1**), which has been described to possess in vitro and in vivo anti-DENV activity [8,9], did not show significant DENV2 activity within the 100 μM concentration range. As per literature [8], amantadine acts at the early stage during the DENV life cycle, most likely during the entry step. The inactivity of amantadine therefore suggests that compounds acting early in DENV life cycle may not be observed as active in this assay. Further studies are necessary to confirm this. However, given the propensity of the adamantane moiety to improve activity and distribution profiles of compounds they are linked to [10], it is likely that the potentiated antiviral activity of compound **3** and **7** is due to the synergistic properties of the adamantane and benzsulfonamide moieties of these hybrids. The DENV2 activity of compounds **3** and **7** suggest, based on our finding with amantadine, that they are active at a later stage during the virus life cycle, most likely through their ability to inhibit DENV2 protease [13], similar to **SF1** and **SF2** [6]. It is also important to note that **3** and **7** may act similarly to amantadine and prevent viral entry because of the inclusion of the adamantane moiety(s) in their structures. This therefore implies that **3** and **7** could present as multifunctional agents that are able to act as anti-DENV agents either during the early or later stage of the virus life cycle. The test compounds did not show any significant cytotoxicity (IC_50_ > 100 µM), indicating that the activity observed for **3** and **7** is a result of their anti-viral activity and not because of their toxicity towards the cell line.

### 2.3. Molecular Modelling

**Figure 4:**
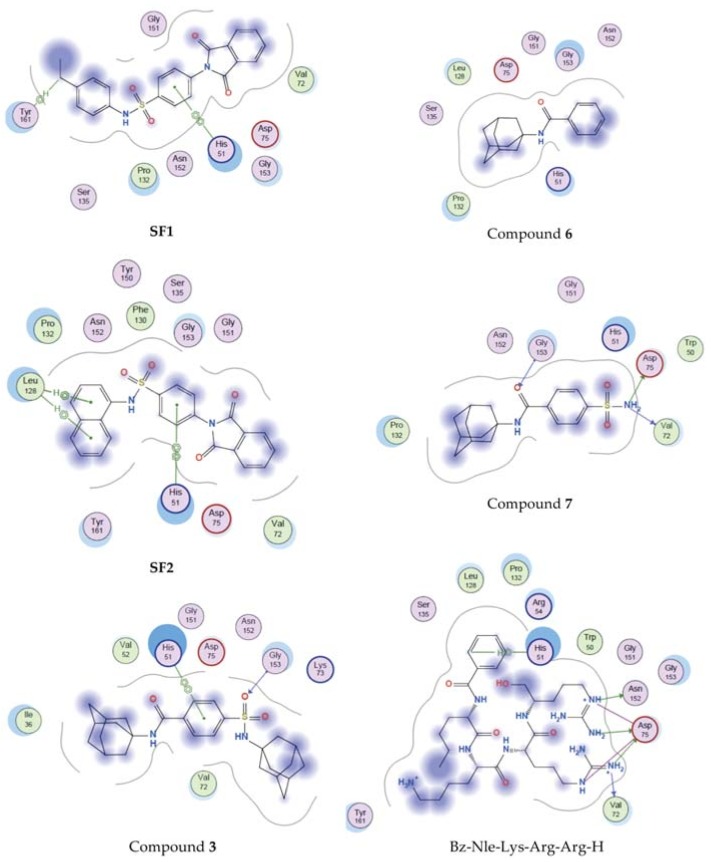
Binding interactions of reference compounds **SF1** and **SF2**, test compounds **3**, **6**, and **7**, and the known substrate inhibitor Bz-Nle-Lys-Arg-Arg-H with the active site of DENV2 NS2B/NS3 protease.

To elucidate the potential mechanism of action of the test compounds molecular docking was used to establish their ability to act as DENV2 NS2B/NS3 protease inhibitors. Compounds **1**, **3**, **6**, and **7** show structural similarities to the known DENV2 NS2B/NS3 protease inhibitors **SF1** and **SF2** [6]; therefore, they are expected to show similar activities. To provide insight, the binding mode of compounds **3**, **6**, and **7** in the DENV2 NS2B/NS3 protease active site were examined with molecular docking using Molecular Operating Environment (MOE) software (Chemical Computing Group, Montreal, Québec, Canada) [14]. Compounds **SF1**, **SF2**, and the known DENV2 NS2B/NS3 protease substrate based inhibitor Bz-Nle-Lys-Arg-Arg-H [15], were used as reference compounds. The structure of DENV2 NS2B/NS3 (PDB entry: 2FOM) [15] was retrieved from the Brookhaven Protein Data Bank (www.rcsb.org/pdb). The active site comprising out of the catalytic triad (His51, Asp75, Ser138) [15] was selected with the help of MOE Site Finder tool and docking was carried out with MOE Dock [14]. Results are shown in Table 3 and Figure 4.

Docking results indicated that as expected the known DENV2 NS2B/NS3 protease inhibitors, **SF1** and **SF2**, were able to form interactions with or are in close proximity to vital residues in the active site of DENV2 NS2B/NS3 protease with binding affinities of −7.835 kcal/mol and −8.011 kcal/mol, respectively. The substrate based inhibitor Bz-Nle-Lys-Arg-Arg-H also showed important interactions with catalytic triad residues with a binding affinity of −11.323 kcal/mol. Compound **6** produced a low binding affinity (−6.124 kcal/mol) and lacked interactions with the active site residues. This may indicate that **6** has no or very weak DENV2 NS2B/NS3 protease inhibitory activity and may also explain the difference in anti-DENV2 activity observed when compared to **3** and **7**. Similar to compound **6**, amantadine (**1**) showed low binding affinity (−4.234 kcal/mol) and did not interact with any residues. This was expected as amantadine is not described as a protease inhibitor. Compound **3** showed similar interactions, binding orientation and binding affinity (−7.413 kcal/mol) as compared to **SF1** and **SF2**. Compound **7** interacted with some comparable residues as Bz-Nle-Lys-Arg-Arg-H with a binding affinity (−7.123 kcal/mol) in the same range as **3**. Both compounds **3** and **7** showed significant anti-DENV2 activity and based on the molecular docking studies part of their mechanism of action may be their ability to inhibit DENV2 NS2B/NS3 protease. It is important to note that, even though these compounds are expected to act as DENV2 NS2B/NS3 protease inhibitors, they may also have additional mechanism(s) of action, and therefore a synergistic effect because of the inclusion of the adamantane moeity. These results therefore justify further investigations into the mechanism of action of compounds **3** and **7**.

## 3. Materials and Methods

### 3.1. Chemistry

#### 3.1.1. General Information

Unless otherwise specified, all of the chemicals were obtained from Sigma-Aldrich or Merck. All of the chemicals were of analytical grade and were used without further purification. The structures of the compounds were characterised by using NMR, Fourier transform infrared (FT-IR), and mass spectrometry (MS) techniques. NMR’s were obtained using a Bruker 400 MHz instrument (Bruker, Coverty, Warwickshire, United Kingdom). The chemical shifts are reported as δ values in ppm downfield from TMS and deuterated residual solvent peaks as internal standards. The coupling constants (*J*) are given in hertz (Hz) and the multiplicities of NMR signals are expressed as: s, singlet; brs, broad singlet; d, doublet; t, triplet; m, multiplet; dd, doublet of doublets; and, dt, doublet of triplets. The ^1^H NMR data is presented as follows: chemical shift in ppm (multiplicity, coupling constant, integration as number of protons). IR spectra were recorded using a Perkin Elmer Spectrum 400 FT-IR/FT-NIR spectrometer (Perking Elmer, Waltham, MA, USA) with an attenuated total reflectance (ATR) attachment. Mass spectra were recorded by Perkin Elmer MS with a Flexar SQ 300 MS detector (Perking Elmer, Waltham, MA, USA) using direct infusion electro-spray ionisation mass spectrometry. High resolution mass spectra (HRMS) were recorded on a Waters API Q-Tof Ultima electro-spray ionisation mass spectrometer (Waters, Milford, MA, USA) at 70 eV and 100 °C. The melting points were determined using a Stuart SMP-10 melting point apparatus (Stuart, Staffordshire, West Midlands, United Kingdom). All of the melting points are uncorrected. Microwave synthesis was performed using a CEM Discover^TM^ (CEM Corporation, Matthews, NC, USA) focused closed vessel (maximum capacity = 30 mL) microwave synthesis system.

#### 3.1.2. Synthesis of *N*-(adamantan-1-yl)-4-[(adamantan-1-yl)sulfamoyl]benzamide (**3**)

*Method A*: 4-(Chlorosulfonyl)benzoic acid (**2**, 0.588 g, 2.7 mmol) in CHCl_3_ (10 mL) was added dropwise to a solution of amantadine hydrochloride (**1**, 0.500 g, 2.7 mmol) and triethylamine (1.484 mL, 10.6 mmol) in CHCl_3_ (10 mL) and stirred for 48 hours at room tempreture. The reaction mixture was concentrated in vacuo to yield a yellow oil, which was purified via flash column chromatography (hexane:ethyl acetate, 70:30) to yield **3** as a white solid, yield: 25%.

*Method B*: 4-(Chlorosulfonyl)benzoic acid (**2**, 0.588 g, 2.7 mmol) in CHCl_3_ (10 mL) was added dropwise to a solution of amantadine hydrochloride (**1**, 1.000 g, 5.4 mmol) and triethylamine (1.484 mL, 10.6 mmol) in CHCl_3_ (10 mL) and under microwave irradiation conditions (100 °C, 150 W, 250 psi) for 30 min. The reaction mixture was concentrated in vacuo to yield a yellow oil, that was purified via flash column chromatography (hexane:ethyl acetate, 70:30) to yield **3** as a white solid, yield: 78%.

Mp: 250 °C. FT-IR (ATR): ν_max_ (cm^−1^) = 3345, 2905, 2851, 1637, 1543, 1453, 1307, 1152, 1090, 747. ^1^H NMR (400 MHz, CDCl_3_): δ 7.93–7.91 (dt, J = 8.4, 2.0 Hz, 2H), 7.82–7.79 (dt, J = 8.4, 2.0 Hz, 2H), 5.87 (NH, brs), 4.68 (NH, brs), 2.13 (s, 9H), 2.00 (s, 3H), 1.77–176 (d, 3H, J = 2.4 Hz), 1.73 (s, 6H), 1.62–1.52 (m, 6H). ^13^C NMR (100 MHz, CDCl_3_): δ 165.3 (C=O), 146.2 (ArC), 139.4 (ArC), 127.4 (2xArCH), 127.1 (2xArC), 55.4 (C-NHCO), 52.8 (C-NHSO_2_), 43.1 (3xCH), 41.6 (3xCH), 36.3 (3xCH_2_), 35.8 (3xCH_2_), 29.47 (3xCH_2_), 29.45 (3xCH_2_). MS (ESI, 70 ev) *m*/*z*: 469 [M + H]^+^. HRMS (ESI/TOF) *m*/*z*: [M + H]^+^ Calcd. for C_27_H_36_N_2_O_3_S 469.2524; Found 469.2525.

#### 3.1.3. Synthesis of *N*-(adamantan-1-yl)benzamide (**6**)

Method A: To a solution of benzoic acid (**4**, 0.390 g, 3.2 mmol) and dimethylformamide (0.031 mL, 0.4 mmol) in dry CH_2_Cl_2_ (30 mL) was added thionyl chloride (0.532 mL, 7.3 mmol). The reaction mixture was subsequently heated at reflux temperature for two hours. After evaporation of the solvent, the residue was dissolved in CHCl_3_ (30 mL) and amantadine hydrochloride (**1**, 0.500 g, 2.7 mmol) was added. Triethylamine (0.742 mL, 5.3 mmol) dissolved in CHCl_3_ (10 mL) was then added dropwise over a period of an hour. The reaction mixture was stirred for a further two hours at room temperature and then diluted with CHCl_3_ (50 mL). The organic fraction was washed with distilled water (3 × 30 mL) and concentrated in vacuo. The residue was purified by flash column chromatography (hexane:ethyl acetate, 70:30) to obtain **6** as a white solid, yield: 25%.

Method: B: To a solution of benzoic acid (**4**, 0.195 g, 1.6 mmol) and dimethylformamide (0.015 mL, 0.2 mmol) in dry CH_2_Cl_2_ (15 mL) was added thionyl chloride (0.266 mL, 3.65 mmol). The reaction mixture was subsequently heated at reflux temperature for two hours. After the evaporation of the solvent, the residue was dissolved in CHCl_3_ (15 mL) and amantadine hydrochloride (**1**, 0.250 g, 1.35 mmol) was added. Triethylamine (0.370 mL, 2.7 mmol) dissolved in CHCl_3_ (5 mL) was added to the reaction mixture and stirred for 30 min. The reaction mixture was then irradiated under microwave conditions (100 °C, 150 W, 250 psi) for 10 min and then diluted with CHCl_3_ (25 mL). The organic fraction was washed with distilled water (3 × 15 mL) and concentrated in vacuo. The residue was purified by flash column chromatography (hexane:ethyl acetate, 70:30) to obtain **6** as a white solid, yield: 64%.

Mp: 155–157 °C. FT-IR (ATR): ν_max_ (cm^−1^) = 2910, 2850, 1716, 1451, 1314, 1260, 1067, 1025, 711. ^1^H NMR (400 MHz, CDCl_3_): δ 7.72–7.70 (m, 2H), 7.48–7.38 (m, 3H), 5.81 (NH, brs), 4.68 (NH, brs), 2.13 (s, 9H), 1.76–169 (m, 6H). ^13^C NMR (100 MHz, CDCl_3_): δ 166.7 (C=O), 136.1 (ArC), 131.0 (ArC), 128.5 (2xArCH), 126.7 (2xArC), 52.3 (C-NH), 41.7 (3xCH), 36.4 (3xCH_2_), 29.5 (3xCH_2_). MS (ESI, 70 ev) *m*/*z*: 256 [M + H]^+^. HRMS (ESI/TOF) *m*/*z*: [M + H]^+^ Calcd. for C_17_H_21_NO 256.1703; Found 256.1701.

#### 3.1.4. Synthesis of *N*-(adamantan-1-yl)-4-sulfamoylbenzamide (**7**)

Method A: To a solution of 4-sulfamoyl benzoic acid (**5**, 0.643 g, 3.2 mmol) and dimethylformamide (0.031 mL, 0.4 mmol) in dry CH_2_Cl_2_ (30 mL) was added thionyl chloride (0.532 mL, 7.3 mmol). The reaction mixture was subsequently heated at reflux temperature for two hours. After evaporation of the solvent, the residue was dissolved in CHCl_3_ (30 mL) and amantadine hydrochloride (**1**, 0.500 g, 2.7 mmol) was added. Triethylamine (0.742 mL, 5.3 mmol) dissolved in CHCl_3_ (10 mL) was then added dropwise over a period of an hour. The reaction mixture was stirred for a further two hours at room temperature and then diluted with CHCl_3_ (50 mL). The organic fraction was washed with distilled water (3 × 30 mL) and then concentrated in vacuo. The residue was purified by flash column chromatography (hexane:ethyl acetate, 50:50) to obtain **7** as a white crystalline solid, yield: 40%.

Method B: To a solution of 4-sulfamoyl benzoic acid (**5**, 0.322 g, 1.6 mmol) and dimethylformamide (0.015 mL, 0.2 mmol) in dry CH_2_Cl_2_ (15 mL) was added thionyl chloride (0.266 mL, 3.65 mmol). The reaction mixture was subsequently heated at reflux temperature for two hours. After evaporation of the solvent, the residue was dissolved in CHCl_3_ (15 mL) and amantadine hydrochloride (**1**, 0.250 g, 1.35 mmol) was added. Triethylamine (0.370 mL, 2.7 mmol) dissolved in CHCl_3_ (5 mL) was then added dropwise over a period of an hour. The reaction mixture was then irradiated under microwave conditions (100 °C, 150 W, 250 psi) for 10 minutes and diluted with CHCl_3_ (25 mL). The organic fraction was washed with distilled water (3 × 15 mL) and then concentrated in vacuo. The residue was purified by flash column chromatography (hexane:ethyl acetate, 50:50) to obtain **7** as a white crystalline solid, yield: 82%.

Mp: 251 °C. FT-IR (ATR): νmax (cm^−1^) = 3348, 2909, 2853, 1648, 1541, 1457, 1328, 1152, 766. ^1^H NMR (400 MHz, CD_3_OD): δ 7.85–7.83 (dt, *J* = 8.7, 2.0 Hz, 2H), 7.77–7.75 (dt, *J* = 8.7, 2.0 Hz, 2H), 2.07–2.06 (d, *J* = 2.8 Hz, 6H), 2.01 (s, 3H), 1.68–1.66 (m, 6H). ^13^C NMR (100 MHz, CD_3_OD): δ 167.27 (C=O), 145.8 (ArC), 139.3 (ArC), 127.6 (2xArCH), 125.8 (2xArC), 52.5 (C-NH), 40.8 (3xCH), 36.11 (3xCH_2_), 29.59 (3xCH_2_). MS (ESI, 70 ev) *m*/*z*: 335 [M + H]^+^. HRMS (ESI/TOF) *m*/*z*: [M + H]^+^ Calcd. for C_17_H_22_N_2_O_3_S 335.1433; Found 335.1430.

### 3.2. Anti-DENV2 Activity Assay

#### 3.2.1. Cells and Viruses

A549 cells (catalog no. CCL-185; ATCC, Manassas, VA, USA) were maintained in Ham’s F-12 medium with 10% fetal bovine serum (FBS) and 1% penicillin-streptomycin at 37 °C in 5% CO_2_. The DENV2 used in this study was prepared by inoculating monolayers of C6/36 cells grown in RPMI 1640 medium with 5% FBS and 1% penicillin-streptomycin. After incubation at 28 °C for four to five days, the cell culture supernatant was collected after clarification of cell debris and was stored at −80 °C until used. 

#### 3.2.2. Cell-Based Flavivirus Immunodetection (CFI) Assay

A549 cells were trypsinised and diluted to a concentration of 2 × 105 cells/mL in culture medium containing 2% FBS. A total of 100 μL of the cell suspension (2 × 104 cells) was dispensed into each well of a 96-well tissue culture plate. The cells were grown overnight in culture medium at 37 °C with 5% CO_2_ and were then infected with the DENV at a multiplicity of infection of 0.3 in the presence of different concentrations of the test compounds for 1 h at 37 °C with 5% CO_2_. The virus inoculum was removed and then replaced with fresh medium containing the test compounds and the cells were incubated at 37 °C with 5% CO_2_ for 48 h. The cells were washed once with phosphate-buffered saline (PBS) and fixed with cold methanol for 10 min. After the fixed cells were washed twice with PBS, they were blocked with PBS containing 1% FBS and 0.05% Tween 20 for 1 h at room temperature. A primary antibody (antibody 4G2) solution was then added, and the mixture was incubated for 3 h. The cells were washed three times with PBS, followed by incubation for 1 h with horseradish peroxidase-conjugated anti-mouse immunoglobulin G. After the cells were washed three times with PBS, 3,3′,5,5′-tetramethylbenzidine substrate solution was added to each well, and the reaction was stopped by adding 0.5 M sulfuric acid. The absorbencies of the solutions were measured with a Tecan Safire II plate reader (Tecan Group Ltd., Männedorf, Switzerland) at 450 nm for viral antigen quantification. Dose-response curves were plotted from the mean absorbance versus the log of the concentration of the test compound. The IC_50_, that is, the concentration of the test compound that inhibited the level of viral envelope protein production by 50%, was calculated by nonlinear regression analysis. All of the experiments were conducted in triplicate.

### 3.3. Cytotoxicity Assay

The cytotoxicities of the test compounds were measured by a Celltiter-Glo Luminescent cell viability assay, according to the manufacturer’s protocol (catalog no. G7570; Promega, Madison, WI, USA). The A549 cell preparation and compound addition were performed, as described above under the “CFI assay”. After 48 h of incubation, the luminescent signals for cells treated with the test compounds were compared to those for cells treated with dimethyl sulfoxide (DMSO) to determine the CC_50_. None of the test compounds showed any cytotoxicity at the maximum concentration tested (100 µM). All of the experiments were conducted in triplicate.

### 3.4. Molecular Modelling

The docking studies were performed using the published DENV NS2B/NS3 protease crystal structure (PDB: 2FOM) [15]. The Molecular Operating Environment (MOE) 2018 software suite [14] was used for the docking studies with the following protocol. (1) The enzyme protein structure was checked for missing atoms, bonds, and contacts. (2) Removal of water molecules, three-dimensional (3D) protonation and energy minimization was carried out with parameters, force field: MMFF94X+solvation, gradient: 0.05, chiral constraint, and current geometry. This minimized structure was used as enzyme for docking analysis. (3) The ligands were constructed using Chemsketch Version 12.01 (Advanced Chemistry Development Inc., Toronto, Ontaria, Canada), saved as mol files, imported to the MOE database, and energy minimized using the MMFF94 force field. (4) Ligands were docked within the DENV NS2B/NS3 protease active site using the MOE Dock application. The active site with the catalytic triad residues (His51, Asp75, Ser135) [15] was selected with the help of the MOE Site Finder tool. The docking algorithm that was chosen for these experiments was based on induced fit docking to allow for flexible interactions of the test ligands with the protein. (5) The best binding pose of each compound was visually inspected and the interactions with the binding pocket residues were analyzed. The selected parameters that were used to calculate the score and interaction of ligand molecules with catalytic triad of DENV NS2B/NS3 were Rescoring function: London dG, Placement: Triangle matcher, Retain: 30, Refinement: Force field, Rescoring 2: London dG. The build-in scoring function of MOE, S-score, was used to predict the binding affinity (kcal/mol) of the ligands with the enzyme protein active site after docking.

## 4. Conclusions

A small series of adamantane sulfamoylbenzamide (**3** and **7**) and adamantane benzamide (**6**) derivatives was synthesized. The synthetic procedures of the final compounds were optimized by substituting the conventional methods with microwave irradiation methods that lead to significant improvements in yields and a reduction in reaction times. The compounds were characterized by ^1^H- and ^13^C-NMR, IR and MS, and a detailed investigation into their structural elucidation was carried out to confirm their chemical structures. Compounds **3** (IC_50_ = 22.4 ± 7.7 µM) and **7** (IC_50_ = 42.8 ± 8.6 µM) showed significant DENV2 inhibitory activities with minimal cytotoxicity (CC_50_ > 100 µM) on A549 cells. Molecular modelling studies revealed that these compounds may act as DENV NS2B/NS3 protease inhibitors, in addition to other potential mechanism(s) of action instilled by the adamantane moiety(ies). Further investigations on these compounds are recommended to explore their potential as agents active against the dengue virus and other viral diseases.

## Figures and Tables

**Table 1:** Reaction conditions and outcomes of the conventional methods compared to the microwave-assisted methods (MWAM).

Compound	Time		% Yields	
	Conventional	MWAM	Conventional	MWAM
**3**	48 h ^1^	30 min ^2^	25 ^3^	78 ^3^
**6**	2 h	10 min	25 ^4^	64 ^4^
**7**	2 h	10 min	40 ^4^	82 ^4^

^1^ A 1:1 ratio of starting materials **1** and **2** was used. ^2^ A 2:1 ratio of starting materials **1** and **2** was used. ^3^ Percantage yield was calculated from the amount of 4-(chlorosulfonyl)benzoic acid (**2**) used. ^4^ Percentage yield was calculated from the amount of amantadine (**1**) used.

**Table 2:** DENV2 activity (µM) and cytotoxicity data (µM) of the test compounds.

Compound	DENV2 IC_50_^a^	CC_50_^b^
1	>100	>100
3	22.4 ± 7.7	>100
6	>100	>100
7	42.8 ± 8.6	>100

^a^ 50% inhibitory concentration values (IC_50_) were determined by the CFI assay. Standard deviations were calculated from at least three independent experiments. ^b^ 50% cytotoxicity values (CC_50_) were determined by the cell viability assay.

**Table 3:** DENV2 NS2B/NS3 protease binding affinity (kcal/mol) and ligand interactions.

Compound	Binding Affinity	Residues Interacting with Ligand
**SF1**	−7.835	His51, Tyr151
**SF2**	−8.011	His51, Leu128
**1**	−4.234	None
**3**	−7.413	His51, Gly153
**6**	−6.124	None
**7**	−7.123	Val72, Asp75, Gly153
Bz-Nle-Lys-Arg-Arg-H	−11.323	His51, Val72, Asp75, Asn152

## Supplementary Materials

The following are available online at http://www.mdpi.com/1420-3049/23/7/1678/s1, ^1^H-NMR, and ^13^C-NMR of compounds **3**, **6** and **7**.
