# Supplementary material for: Microwave Optimized Synthesis of N-(adamantan-1-yl)-4-[(adamantan-1-yl)-sulfamoyl]benzamide and Its Derivatives for Anti-Dengue Virus Activity
Source: Molecules. 2018 Jul 10;23(7):1678. doi: 10.3390/molecules23071678 (PMC6099921; doi:10.3390/molecules23071678)
Supplement: Supplementary file 1 [file molecules-23-01678-s001.pdf]

Synthesis and evaluation of *N*-(adamantan-1-yl)-4-[(adamantan-1-yl)-sulfamoyl]benzamide and its derivatives as anti-dengue virus agents

Jacques Joubert [1,*], Eugene B. Foxen [1] and Sarel F. Malan [1]

[1] *Pharmaceutical Chemistry, School of Pharmacy, University of the Western Cape, Private Bag X17, Bellville, South Africa*; jjoubert@uwc.ac.za; efoxen@uwc.ac.za; sfmalan@uwc.ac.za

* Correspondence: jjoubert@uwc.ac.za; Tel.: +27-21-959-2195

Supplementary information

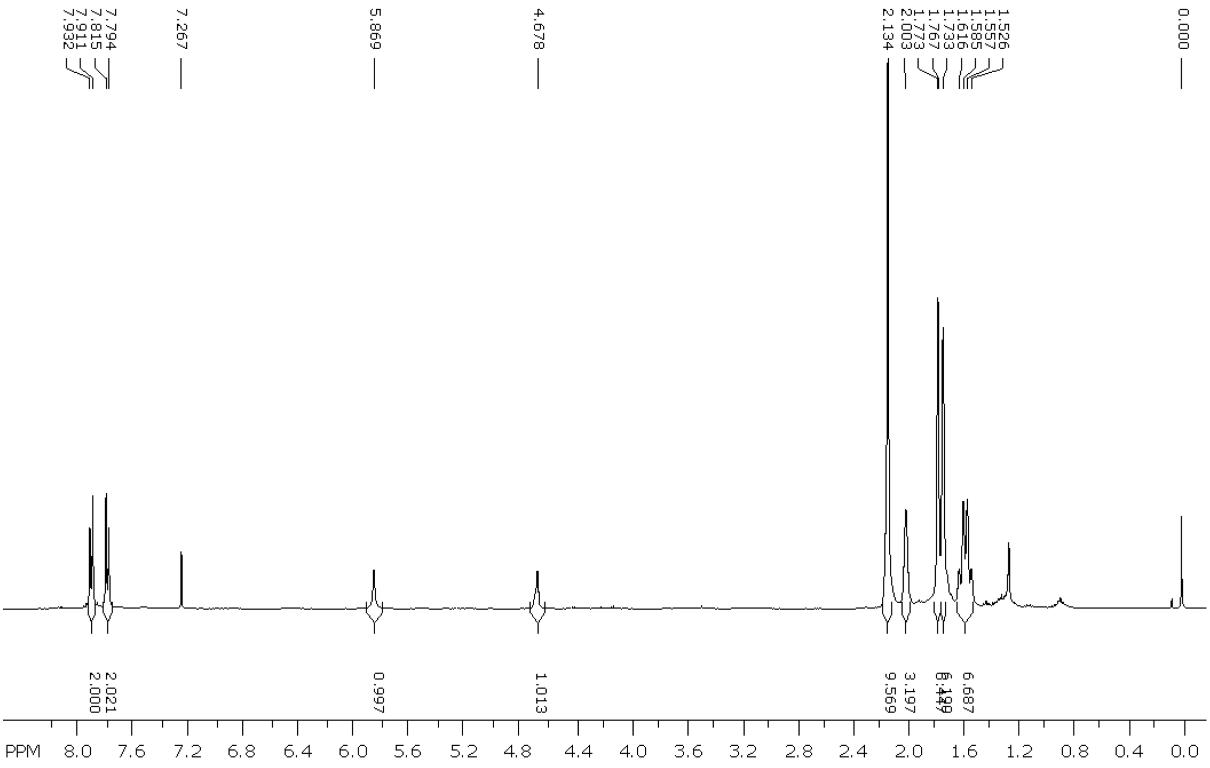

Supplementary Figure 1. ^{1}H-NMR of **3**.

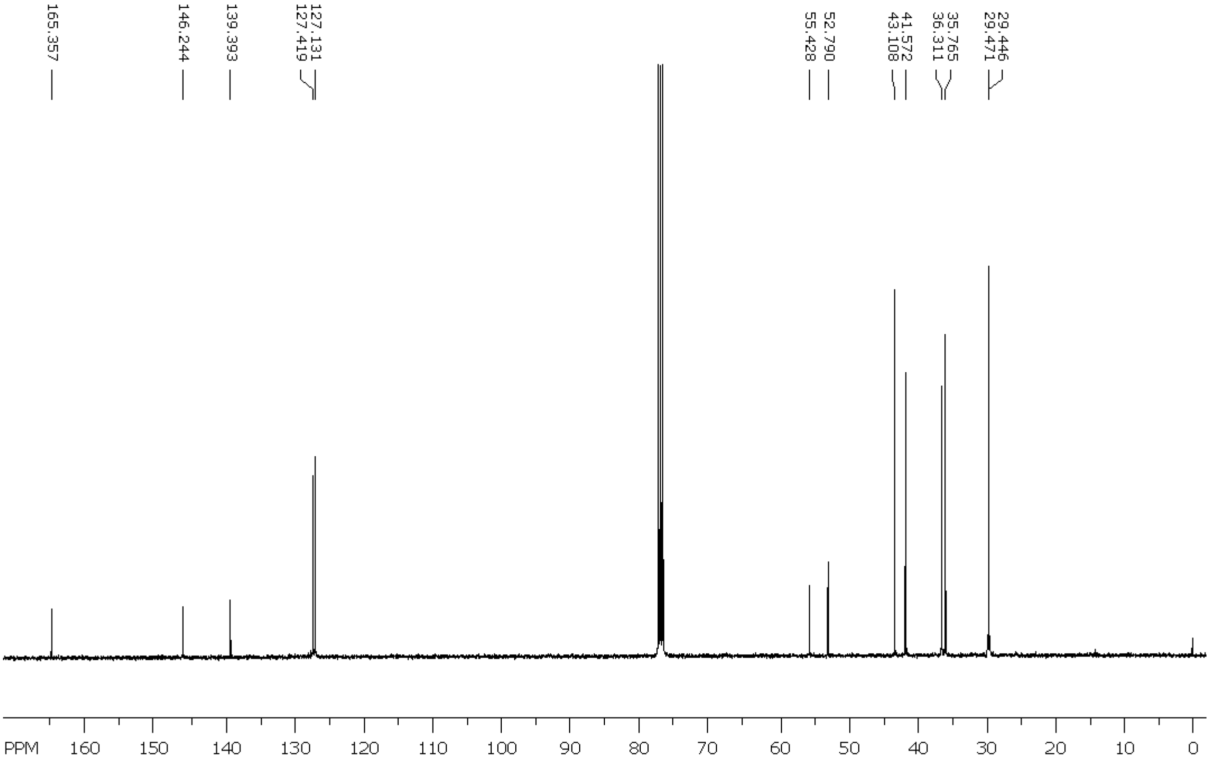

Supplementary Figure 2. ^{13}C-NMR of **3**.

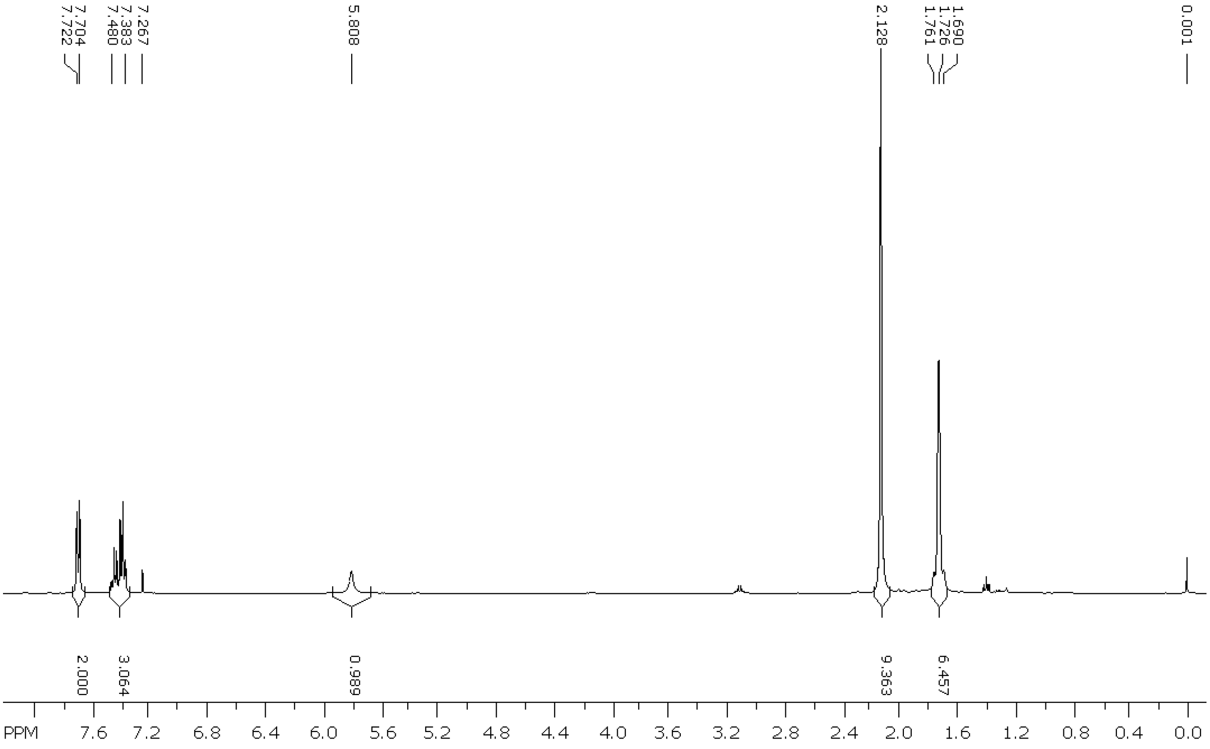

Supplementary Figure 3. ^{1}H-NMR of **6**.

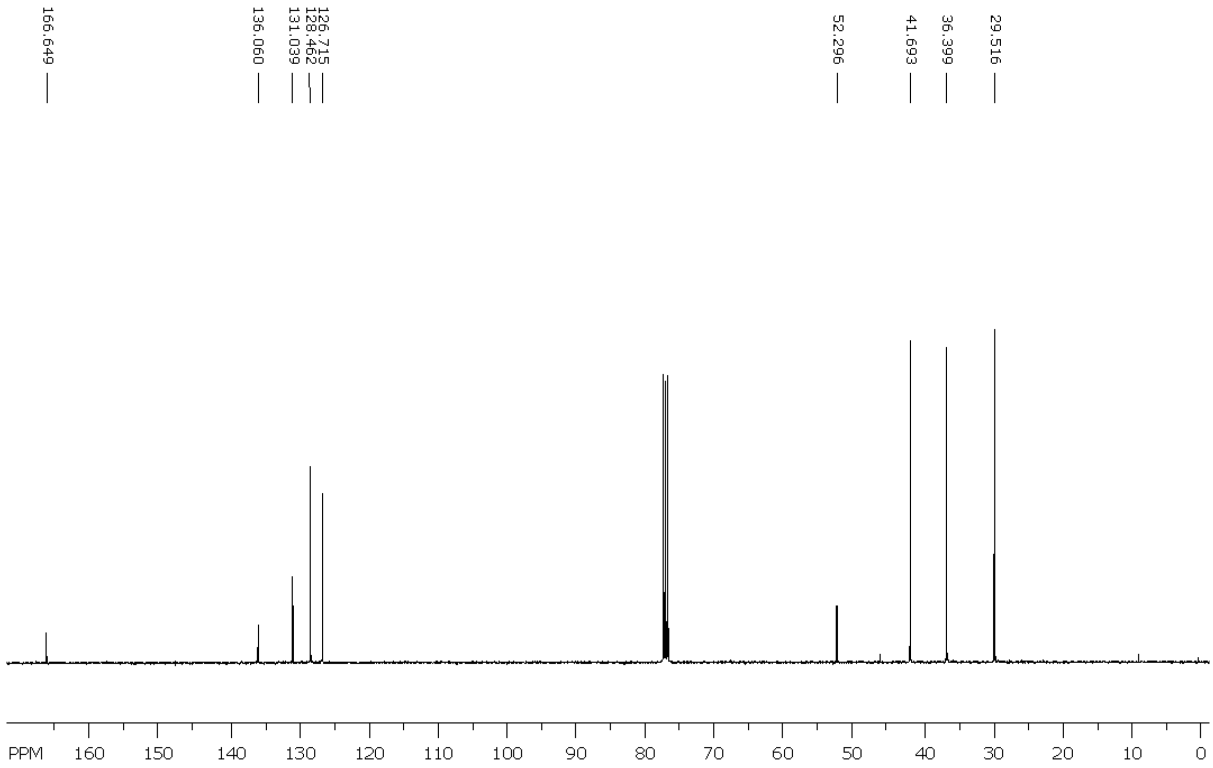

Supplementary Figure 4. ^{13}C-NMR of **6**.

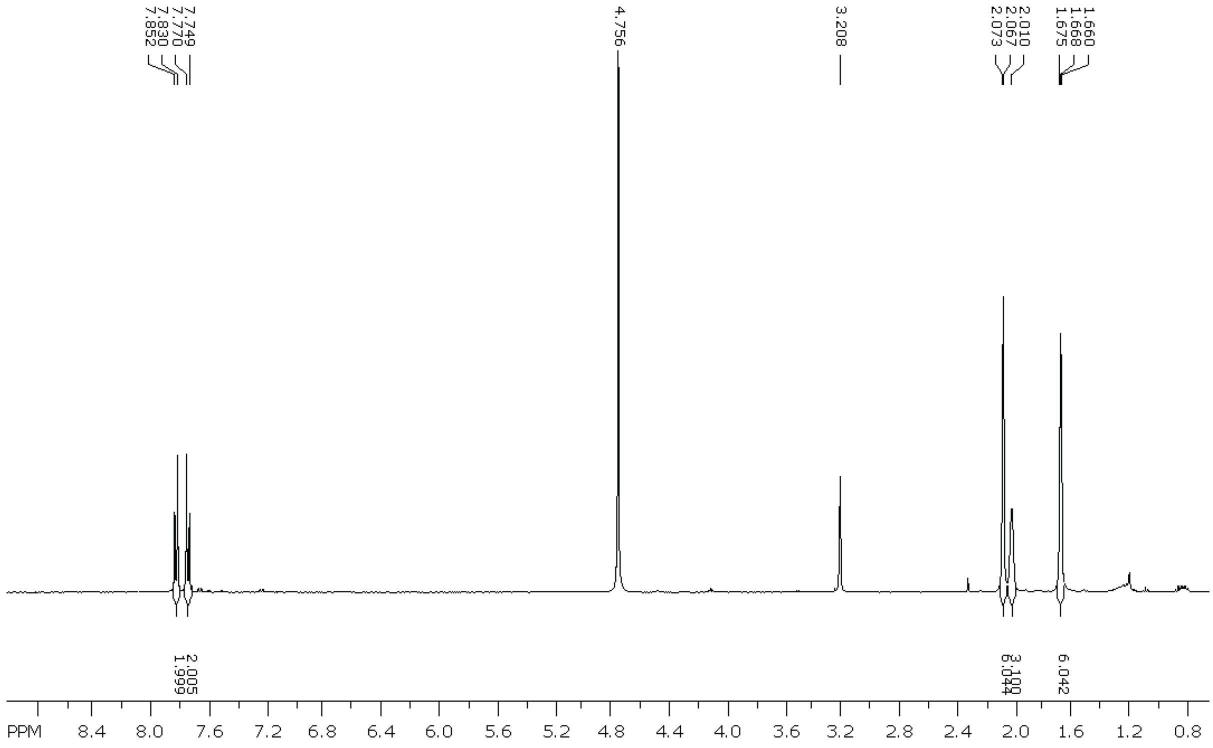

Supplementary Figure 5. ^{1}H-NMR of **7**.

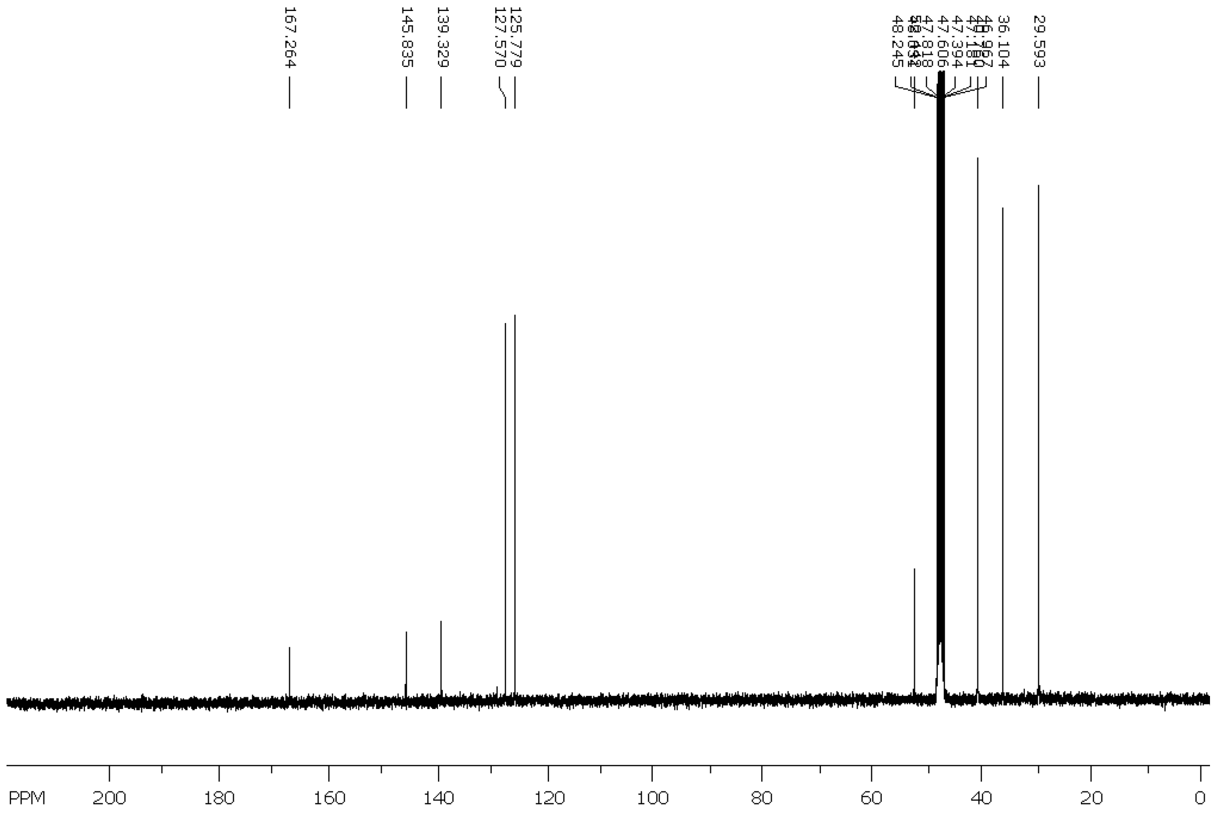

Supplementary Figure 6. ^{13}C-NMR of **7**.
